# Evaluation of personalised, one-to-one interaction using Montessori-type activities as a treatment of challenging behaviours in people with dementia: the study protocol of a crossover trial

**DOI:** 10.1186/1471-2318-10-3

**Published:** 2010-01-24

**Authors:** Eva S van der Ploeg, Daniel W O'Connor

**Affiliations:** 1 Aged Mental Health Research Unit, Monash University, Melbourne, Australia; 2 Kingston Centre, Warrigal Road, Cheltenham, Vic 3192, Australia

## Abstract

**Background:**

The agitated behaviours that accompany dementia (e.g. pacing, aggression, calling out) are stressful to both nursing home residents and their carers and are difficult to treat. Behaviours stemming from pain, major depression or psychosis benefit from treatment with analgesics, antidepressants or antipsychotics. In other cases, psychotropic medications have limited efficacy but are used very widely. Therefore, increasingly more attention has been paid to nonpharmacological interventions which are associated with fewer risks. The aim of the current study is to test if personalised one-to-one interaction activities based on Montessori principles will reduce the frequency of behavioural symptoms of dementia significantly more than a relevant control condition.

**Methods/Design:**

We will conduct a controlled trial with randomised cross-over between conditions. Persons with moderate to severe dementia and associated behavioural problems living in aged care facilities will be included in the study. Consented, willing participants will be assigned in random order to Montessori or control blocks for two weeks then switched to the other condition. Montessori activities derive from the principles espoused by Maria Montessori and subsequent educational theorists to promote engagement in learning, namely task breakdown, guided repetition, progression in difficulty from simple to complex, and the careful matching of demands to levels of competence. The control intervention consists of conversation or reading from and looking at pictures in a newspaper to control for non-specific benefits of one-to-one interaction. Presence of target behaviour will be noted as well as level of engagement and type of affect displayed. Secondary measures also include the Cohen-Mansfield Agitation Inventory and information on time and funds spend to prepare the activities.

**Discussion:**

If our results show that use of Montessori activities is effective in treating challenging behaviours in individuals with dementia, it will potentially provide a safer and more enjoyable intervention rather than reliance on pharmacology alone.

**Trial Registration:**

Australian New Zealand Clinical Trials Registry - ACTRN12609000564257

## Background

Dementia is associated with cognitive decline as well as behavioural and psychological symptoms (BPSD). Agitation (e.g. pacing and calling out) is the most commonly exhibited symptom in older adults with dementia [1,2]. The prevalence of agitated behaviours amongst persons with dementia in nursing homes is 48-82% [3]. These challenging behaviours are stressful to carers and difficult to treat. Behaviours stemming from pain, major depression or psychosis benefit from treatment with analgesics, antidepressants or antipsychotics. In other cases, psychotropic medications have limited efficacy but are widely used [4]. Some pharmacological interventions are reported to precipitate agitation in nursing home populations [5-7] and can have adverse effects including confusion, somnolence, gait abnormalities and falls [8]. Therefore, increasingly more attention has been paid to nonpharmacological interventions which are associated with fewer risks [9].

Three psychologically-oriented paradigms have emerged to explain BPSD and to generate testable interventions. Learning theory asserts that behaviours are reinforced when carers reward them with attention. Calling out, for example, increases in frequency if nursing staff attend to residents when they are noisy but otherwise ignore them [10]. In the *unmet needs *paradigm, inappropriate behaviours stem from normal human needs - physical, emotional and social - that carers fail to perceive and address [11]. Needs for social interaction and physical movement, for example, might be addressed by carefully selected group activities and exercise. According to the *stress threshold model*, dementia reduces the capacity to cope with stress, resulting in inappropriate behaviours [12]. Stress levels can be modulated to tolerable levels by attending to signals of distress and alternating periods of rest and activity. In reality, most psychosocial treatments blend elements of all three paradigms.

Recent reviews and meta-analysis [13-15] showed that sensory interventions (e.g. aromatherapy and hand massage), one-to-one social interaction, individualised music, recreation therapy and family videotapes reduced BPSD more compared to conditions offering an equivalent level of social interaction in a small number of robustly designed studies with moderate to strong quality ratings and adequate statistical precision [14,15]. The largest effect sizes were found in studies of treatments that could be tailored to participants' backgrounds, interests and skills. For example, music that participants had enjoyed earlier in life reduced agitation better than standard classical music [16] while audiotapes of a family member's voice worked better than a stranger's voice [17]. A few treatment effect sizes reached 0.7, exceeding those of most psychotropic medications [14,15]. The calibre of research was generally low, however, and only 17 of 37 carefully selected studies from a total of 176 had "positive" results. Most studies were flawed by low symptom counts, unreliable behaviour ratings, small sample size and limited time-frames. Few of them controlled for the benefits of the one-to-one interaction that underpins most psychosocial treatments. Agitation can respond well to real, and even simulated, personal contact [17,18] making control conditions providing equivalent social attention, interaction and diversion desirable.

Taking these limitations in account there still is evidence that one-to-one interaction with a diversional or ADL focus outperforms baseline or "usual care" conditions in reducing agitation and improving affect. Activities based on Montessori principles might perform even better. A recent study using a Montessori-based activities program (including 5 pre-selected categories of activities) showed that the Montessori program resulted in improvement in aggressive and physically non-aggressive behaviours, an increase of positive affect and fewer difficulties in providing care compared to a "presence"-condition [19].

Montessori-based activities derive from the principles espoused by Maria Montessori and subsequent educational theorists to promote engagement in learning, namely task breakdown, guided repetition, progression in difficulty from simple to complex, and the careful matching of demands to levels of competence [20,21]. With respect to dementia, Montessori activities provide socialisation, meaningful activity and diversion through the medium of one-to-one interaction, in line with Cohen-Mansfield's theory of unmet need [11], and they can easily be adapted to the interests and skills of people with dementia. Activities are designed to tap procedural memory which is better preserved than verbal memory while minimising language demands and providing external cues to compensate for cognitive deficits. Familiar objects provide cues to their own use (e.g. playing cards suggest sorting them in a sequence) and tasks are demonstrated by a facilitator who then hands the object to participants, thus prompting them to follow suit.

The current study explores the effect of personalised one-to-one interaction based on Montessori principles on BPSD in residents in aged care facilities compared to a plausible control condition, which controls for the benefits of the one-to-one interaction that accompanies the Montessori intervention. To this end, we will conduct a controlled trial with randomised cross-over between conditions to test the hypothesis that individualised, goal-directed activities reduce the frequency of behavioural symptoms of dementia significantly and increase positive affect and engagement more than a relevant control condition.

## Methods/Design

### Study design

We will use an efficient, economical design with random allocation to treatment or control conditions followed by cross-over. Repeated measures RCTs minimise intra- and inter-individual differences since all participants are subject to both conditions and behaviours vary greatly in frequency within and between people with dementia from hour to hour and day to day [14].

### Ethical considerations

The protocol has been approved by the ethics committees of Monash University and all the health organisations to which the participating aged care facilities (ACFs) are affiliated (Southern Health, Peninsula Health and Alfred Health).

It is most unlikely that participants can provide informed consent. In Victoria, the "person responsible" (usually a spouse or child) consents to the participation of non-competent persons with dementia in non-invasive studies. While the person responsible provides written, informed consent to the study, persons with dementia must assent to participation, as shown by their willingness to participate in the activities.

### Setting

The study will be conducted in mainstream and psychogeriatric ACFs in south-east Melbourne. Preference will be given to larger facilities (≥60 beds).

### Recruitment

Aged care facilities will be contacted by telephone to explain the studies. If ACFs show interest in the study, researchers will visit the facility to provide all relevant parties (director of nursing, nursing staff and diversional therapists) with more detailed written and oral information. If facilities consent to participate in the study a pre-selection screening is done to identify eligible participants. An appointed delegate of the ACF will then contact the person responsible (PR) for each participant to ask if they consent with forwarding their contact details to a Monash researcher. When verbal consent is given a senior researcher will contact the PR to explain the studies and answer queries. If a PR expresses interest in the studies they are send a Participant Information and Consent Form package, that includes detailed information on the study procedure, a consent form and pre-paid addressed return envelop. Once written consent is received, researchers gather baseline information to confirm eligibility of the person before enrolment in the study.

### Participants

Evidence suggests that verbally disruptive behaviours (e.g. calling out) link more with lowered mood while physically disruptive behaviours (e.g. pacing) link more to a lack of meaningful occupation and socialisation [11]. While these types of behaviours often occur together, there is a trend to distinguish between them for research purposes. The current study focuses on physically disruptive behaviours, because they are more common in ACFs. We also expect Montessori-activities to have more effect on physical behaviours, because the activities may reduce the lack of meaningful occupation and socialisation. Selected participants must therefore display at least one physically agitated behaviour that occurs daily at times other than during nursing interventions and to a degree that requires staff intervention. The target behaviour will be selected per participant in discussion with nursing staff based on frequency, severity and capacity using the Cohen Mansfield Agitation Inventory [22].

#### Inclusion criteria

(1) A chart diagnosis of moderate to severe dementia.

(2) Standard cognitive tests are invalid in this group since most participants will be severely impaired. Many will score zero on the widely-used Mini-Mental State Examination [23]. The chart diagnosis of dementia will additionally be confirmed by interviewing staff with the Clinical Dementia Rating [24].

(3) At least one behavioural symptom as defined above.

(4) An assessment by the ACF staff, GP and/or psychiatrist that behaviours are not due primarily to untreated or inadequately treated pain, physical illness, major depression or psychosis.

(5) Residence in a high care, or mixed high care and low care ACF for three or more months.

(6) Consent to the study by the PR as defined by the Victorian Civil and Administrative Tribunal.

#### Exclusion criteria

(1) Active treatment that might change over the study period by a psychiatrist or aged mental health team.

(2) A current, acutely life-threatening physical illness as reported by ACF staff and the GP.

(3) Behaviours that present a hazard to researchers (e.g. unpredictable aggression).

(4) No detectable verbal or non-verbal response to the presence of another person verified by a researcher on two occasions.

### Interventions

Treatments should be applied over a sufficient number of days to account for random "noise" but not extend for so long that participation is compromised by disease progression, inter-current illness and unavoidable changes to psychotropic medications. Our selected period of four weeks is a reasonable compromise. Similarly, individual sessions should be long enough to detect real changes in behaviour before, during and after sessions but not so long that observations are likely to be interrupted by meals, nursing interventions and visitors. Ninety minutes is the longest feasible period in our experience. Consented, willing participants will be assigned in random order to Montessori or control blocks for two weeks then switched to the other condition. Interventions, whether Montessori or control, will be delivered twice weekly at times when nursing staff report that target behaviours are most likely to be present (excluding times of personal nursing care). Interventions (Montessori or control) will be delivered for 30 minutes per session. Observations will be made for 30 minutes before, 30 minutes during, and 30 minutes after interventions, giving a total observation period of 90 minutes per session, and 12 hours overall per person.

#### Montessori intervention

Montessori activities derive from the principles espoused by Maria Montessori and subsequent educational theorists to promote engagement in learning, namely task breakdown, guided repetition, progression in difficulty from simple to complex, and the careful matching of demands to levels of competence [20,21]. With respect to dementia, activities are designed to tap procedural memory which is better preserved than verbal memory while minimising language demands and providing external cues to compensate for cognitive deficits. Suitable activities come in dozens of varieties and can be shaped to former interests and skills. They range from simple tasks (e.g. sorting cards) to more complex ones (e.g. making puzzles from familiar photographs) but none are very difficult. Facilitators give participants choices, demonstrate what is required and encourage success [25]. The researchers will select up to 10 Montessori-type activities per participant [20-25] based on discussion with family members about their former interests and hobbies. Charts will be checked to identify health factors that may impede on some of the activities (e.g. visual or hearing impairment). On treatment days, the facilitator will seek to engage the resident in one or more of the selected activities, with flexibility to respond to residents' perceived interests. Where engagement proves impossible, facilitators will remain with participants and follow whatever they do for the required period.

#### Control intervention

We wish to control for the non-specific benefits of the one-to-one interaction implicit in many psychosocial treatments while recognising that Montessori-type activities are themselves reliant on personal contact. The control condition must therefore offer significant interaction minus the core Montessori elements of personalisation, graded difficulty, cueing and task demonstration coupled with minimal language demands. Based on earlier work in which a neutral control condition of audio taped readings from a textbook significantly reduced physical agitation [17], the control condition will consists of facilitators engaging participants in verbal interaction by means of general conversation, reading from or looking at pictures in a newspaper. This control condition offers equivalent personal attention, is practicable and can be replicated in future studies. No special provision will be made for participants from non-English speaking backgrounds, in line with real-world conditions in most ACFs and with our requirement for a non-individualised control. As before, where engagement proves impossible, facilitators will remain with participants and follow whatever they do for the required period.

### Outcomes measures

A discretely positioned, trained researcher will record if the selected physically agitated behaviour is present or absent at one-minute intervals over the three 30-minute observation periods giving 30 data points per period and 90 per session. Behaviour counts will range from zero to 30 per period. This method was used successfully in previous studies with very high levels of inter-rater reliability [17,26-29].

The *primary *measure in this trial will be the change in mean counts of the target physically agitated behaviour across before, during and after intervention phases.

*Secondary *measures will include:

(a) Rating scales completed by the observing researcher during the 30 minute treatment periods at one-minute intervals of the participant's predominant levels of engagement and affect.

We distinguish between four types of engagement based on the Menorah Park Engagement Scale [30]: 1) non-engagement, which is described as a blank stare or engagement with something else than the facilitator and/or the presented activity; 2) self-engagement, engagement with the self, including engagement displayed when a person shows agitated behaviours, e.g. wandering, repetitious behaviour (wringing hands); 3) passive engagement, the person is watching or listening to the facilitator or activity; 4) constructive engagement, the person is actively involved with the facilitator or activity, e.g. by speaking to the facilitator or handling materials used in the activity (folding towels or leafing through a book).

For each minute the predominant type of affect is noted. We will include three positive emotions (pleasure, contentment and interest), one neutral and three negative emotions (anger, sadness and fear/anxiety).

(b) The 29-item Cohen-Mansfield Agitation Inventory [22] completed by the researcher in discussion with nursing staff in closest contact with the resident at the end of each two-week period, relating to behaviour in the preceding fortnight. Where possible, the same staff members will be questioned on each of the four occasions.

(c) A global rating by the residential managers of satisfaction with the outcome of the trial.

(d) The time and costs involved in preparing Montessori activities.

### Reliability, training and supervision

It is impossible to blind researchers to Montessori or control conditions. Physical behaviours like pacing can be monitored using pedometers but such methods are not ideal in this study since target behaviours vary from one participant to another. Instead, we will rely on the behaviour counts recorded by well trained and supervised observers. Researchers will co-rate behaviours under supervision by an experienced observer until they reach a *kappa *inter-rater agreement level of ≥0.8. Based on past experience, we expect this will take one day of training in a nursing home. Inter-rater reliability will again be checked later on in the project.

Activity facilitators will receive extensive coaching in the theory and practice of Montessori-type activities in a nursing home setting. The project manager (EvdP) will model treatment and control interventions together with ways of relating generally to elderly people with advanced dementia. After a generic training day, individual facilitators will do training sessions with persons with dementia under supervision of the project manager. At the start of the project (and for as long as necessary) facilitators will discuss the outcomes of the interview with the relative, the possible activities per resident and each Montessori and control session with the project manager.

### Sample size calculation

Sample size was calculated for the primary outcome measure (physically agitated behaviour) for a two-sided hypothesis test with a Type I error rate of 0.05 and a Type II error rate of 0.10 (90% power). In the 30-minute baseline observation period, we anticipate that participants will display a mean count of 5.0 physically agitated behaviours (maximum 30) with a standard deviation of 2.15. We anticipate a mean improvement of 0.69 behaviours per period during the control condition (effect size 0.32) compared with a mean improvement of 1.53 per period during Montessori activities (effect size 0.71), giving a difference of 0.39 between the conditions. Based on data from the study by Garland *et al*. [17], we estimate a within-person correlation of 0.70. A sample of 70 participants will be required to detect this difference, assuming the same variance (SD = 2.15) across conditions. Based on previous experience, and the relatively brief duration of this study, we expect 10% attrition and so will recruit 80 participants.

### Statistical analysis

We will use two-way repeated measures analysis of variance to test the significance of changes in the number of physically agitated behaviours over time (before, during, after treatment). We will use simple main effects to determine the specific effect of the Montessori intervention relative to the control condition and we will test for the interaction between treatment and time. Simple contrasts will be used to tease out more detailed relationships, including testing the primary hypothesis that Montessori activities reduce the frequency of physically agitated behaviours significantly more than a plausible control condition.

Analysis will be by intention to treat. Baseline characteristics of participants who drop out during the study will be compared to those who complete it to assess patterns of loss to follow-up and provide insights into the degree to which results can be generalised.

## Discussion

The proposed study will help meet the need for better controlled trials of psychosocial treatments. Our findings will guide family and professional carers in their selection of available evidence-based ways to reduce stressful behavioural symptoms that respond only partially to psychotropic medications. If our results show that use of Montessori activities is effective in treating challenging behaviours in individuals with dementia, it will potentially provide a safer and more enjoyable intervention rather than reliance on pharmacology alone. The activities can be applied by family carers, ACF staff and volunteers in a very wide range of settings.

## Competing interests

The authors declare that they have no competing interests.

## Authors' contributions

DOC designed the original study, EvdP assisted in working out the exact procedure. EvdP is project manager of the study. Both authors contributed to the writing of this paper; and read and approved the final version of the manuscript.

## Pre-publication history

The pre-publication history for this paper can be accessed here:

http://www.biomedcentral.com/1471-2318/10/3/prepub
